# Genome sequence of the olive tree, *Olea europaea*

**DOI:** 10.1186/s13742-016-0134-5

**Published:** 2016-06-27

**Authors:** Fernando Cruz, Irene Julca, Jèssica Gómez-Garrido, Damian Loska, Marina Marcet-Houben, Emilio Cano, Beatriz Galán, Leonor Frias, Paolo Ribeca, Sophia Derdak, Marta Gut, Manuel Sánchez-Fernández, Jose Luis García, Ivo G. Gut, Pablo Vargas, Tyler S. Alioto, Toni Gabaldón

**Affiliations:** CNAG-CRG, Centre for Genomic Regulation (CRG), Barcelona Institute of Science and Technology (BIST), Baldiri i Reixac 4, 08028 Barcelona, Spain; Universitat Pompeu Fabra (UPF), 08003 Barcelona, Spain; Bioinformatics and Genomics Department, Centre for Genomic Regulation (CRG), The Barcelona Institute of Science and Technology, Dr. Aiguader 88, Barcelona, 08003 Spain; Universitat Autònoma de Barcelona, Barcelona, Spainᅟ; Royal Botanical Garden of Madrid. Consejo Superior de Investigaciones Científicas (CSIC), 28014 Madrid, Spain; Departamento de Biología Ambiental, Centro de Investigaciones Biológicas (CIB). Consejo Superior de Investigaciones Científicas (CSIC), 28040 Madrid, Spain; Paisajismo. Área Corporativa de Inmuebles. Grupo Santander, Boadilla del Monte, Madrid Spain; Institució Catalana de Recerca i Estudis Avançats (ICREA), Pg. Lluís Companys 23, 08010 Barcelona, Spain; CRG-Centre for Genomic Regulation, Doctor Aiguader, 88, 08003 Barcelona, Spain; Centre Nacional d’Anàlisi Genòmica (CNAG-CRG, Baldiri Reixac, 4, 08028 Barcelona, Spain; Pablo Vargas. Royal Botanical Garden of Madrid, Plaza de Murillo 2, 28014 Madrid, Spain

**Keywords:** Olive tree genome, Genomics, Assembly, Annotation

## Abstract

**Background:**

The Mediterranean olive tree (*Olea europaea* subsp. *europaea*) was one of the first trees to be domesticated and is currently of major agricultural importance in the Mediterranean region as the source of olive oil. The molecular bases underlying the phenotypic differences among domesticated cultivars, or between domesticated olive trees and their wild relatives, remain poorly understood. Both wild and cultivated olive trees have 46 chromosomes (2n).

**Findings:**

A total of 543 Gb of raw DNA sequence from whole genome shotgun sequencing, and a fosmid library containing 155,000 clones from a 1,000+ year-old olive tree (cv. Farga) were generated by Illumina sequencing using different combinations of mate-pair and pair-end libraries. Assembly gave a final genome with a scaffold N50 of 443 kb, and a total length of 1.31 Gb, which represents 95 % of the estimated genome length (1.38 Gb). In addition, the associated fungus *Aureobasidiumpullulans* was partially sequenced. Genome annotation, assisted by RNA sequencing from leaf, root, and fruit tissues at various stages, resulted in 56,349 unique protein coding genes, suggesting recent genomic expansion. Genome completeness, as estimated using the CEGMA pipeline, reached 98.79 %.

**Conclusions:**

The assembled draft genome of *O. europaea* will provide a valuable resource for the study of the evolution and domestication processes of this important tree, and allow determination of the genetic bases of key phenotypic traits. Moreover, it will enhance breeding programs and the formation of new varieties.

## Data description

### Sequencing

Genomic DNA was extracted from leaf tissue of a single Mediterranean olive tree (*Olea europaea* L. subsp. *europaea* var. *europaea cv. 'Farga'*; NCBI Taxonomy ID: 158383). This tree, named ‘Santander’, was translocated from the Maestrazgo region (Eastern Spain) to Boadilla del Monte (Madrid, Spain) in 2005. *O. europaea* is a common tree in Spain and there are no legal restrictions on its use for research, including cv. Farga.

The tree age was estimated to be 1,200 years old based on dendrometric analyses (Antonio Prieto-Rodríguez personal communication). A combination of fosmid and whole genome shotgun (WGS) libraries were sequenced using Illumina sequencing equipment.

The standard Illumina protocol was followed, with minor modifications to create short-insert paired-end (PE) libraries (Illumina Inc., Cat. # PE-930–1001), which were run on different types of Illumina sequencers (MiSeq 2×250, 2×300, 2×500, 1×600 and HiSeq2500 2×150) according to standard procedures. The MiSeq XL modes (2×500 and 1×600) were carried out according to the MiSeq modifications reported in [1] and with the technical support of Illumina.

Primary data analysis was carried out using the standard Illumina pipeline (HCS 2.0.12.0, RTA 1.17.21.3). Mate-pair (MP) libraries (3, 5, 7 and 10 kb fragment sizes) were constructed at the CRG sequencing unit according to the Nextera Mate Pair Preparation protocol (Illumina Inc.), and sequenced on the HiSeq2500 platform in 2x150bp read length runs. The number of lanes and raw sequenced outputs for each library are summarized in Table 1.

**Table 1:** Sequencing libraries and respective yields used for whole genome shotgun sequencing and fosmid pools

Library	Mode	Name	Yield (Gb)
PE400	2*262	837G_B	8.3
PE400	2*312	837G_B	68.0
PE400	2*255	837G_B	8.2
PE560	2*312	846G_D	33.9
PE560	2*151	846G_D	99.2
PE560	2*500	846G_E_PCR	14.1
PE560	2*151	846G_E_PCR	46.8
PE725	2*151	837G_E_PCR	96.3
PE725	1*625	837G_E_PCR_2	15.2
MP3k	2*151	T587	33.9
MP5k	2*151	T586	40.3
MP7k	2*151	T585	37.6
MP10k	2*151	T584	42.7
FP PE350	2*151	1FP to 96FP	11.3*

*mean yield

**Table 2:** Summary statistics of the Oe6 assembly

Oe6Assembly	Length (bp)	Contiguity (bp)			Completeness (CEGMA)	
		N10	N50	N90	Complete	Partial
Contigs	1,264,682,749 (59,457)	138,917 (695)	52,353 (7,085)	11,476 (25,802)	−	−
Scaffolds	1,318,652,350 (11,038)	1,088,680 (94)	443,100 (901)	110,965 (3099)	98.8 %	98.8 %

Numbers of contigs/scaffolds are shown in parentheses

**Fig. 1:**
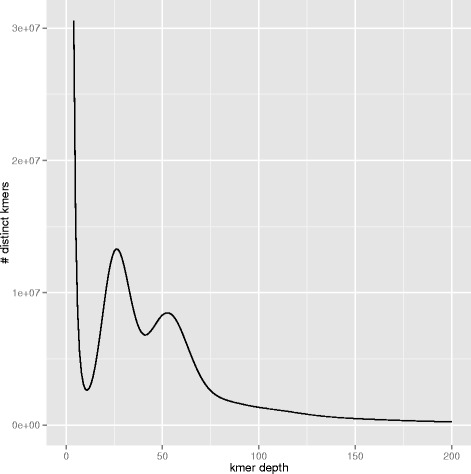
Kmer spectrum. Using Jellyfish v1.1.10, 17-mers were counted in a subset of whole genome shotgun paired-end reads corresponding to the PE560 2x150 sequencing run. The density plot of the number of unique kmer species (y axis) for each kmer frequency (x axis) is plotted. The homozygous peak is observed at a multiplicity (kmer coverage) of 52 x, while the heterozygous peak is observed at 26 x. The tail extending to the right represents repetitive sequences. The total number of kmers present in this subset was 71,902,584,399. From these data, the Genome Character Estimator (gce) estimates the genome size to be 1.32 Gb

Preliminary kmer analysis of PE data (Fig. 1) indicated a high level of heterozygosity in this sample. To reduce the risk of separately assembling two different haplotypes from the same locus and including them in the final assembly, a fosmid pooling strategy was chosen similar to the one used for the oyster genome project [2]. A fosmid library of 155,000 clones was constructed in the pNGS vector (Lucigen Corp.). Ninety-six pools of ~1,600 clones each were made, and the purified DNA was used to construct short-insert PE libraries using the TruSeq™ DNA Sample Preparation Kit v2 (Illumina Inc.) and the KAPA Library Preparation kit (Kapa Biosystems) according to manufacturers’ instructions. The pools were sequenced using TruSeq SBS Kit v3-HS (Illumina Inc.), in PE mode, 2×150 bp, in a fraction of a sequencing lane of the HiSeq2000 flowcell v3 (Illumina Inc.) according to standard Illumina operation procedures. The raw sequence yield per pool was 11.3 Gb on average (SD: 2 Gb), corresponding to ~150 × depth. In addition a fosmid-end library was created from the same set of clones using the Lucigen pNGS protocol and run in one lane of a HiSeq2000.

RNA was prepared from seven different tissues or developmental stages (root, young leaf, mature leaf, flower, flower bud, immature fruit, and green olives), using the Zymo ZR Plant RNA extraction kit (Zymo Research, Irvine, CA). Then, RNA-Seq libraries were prepared using the TruSeq™ RNA Sample Prep Kit v2 (Illumina Inc.) with minor modifications, and libraries were sequenced using the TruSeq SBS Kit v3-HS in PE mode with a read length of 2×75 bp. Over 50 million PE reads per sample were generated in a fraction of a sequencing lane on a HiSeq2000 (Illumina Inc.), following the manufacturer’s protocol. Image analysis, base calling and run quality scoring were processed using the manufacturer’s software Real Time Analysis (RTA 1.13.48), followed by generation of FASTQ sequence files using CASAVA software (Illumina Inc.).

### Genome assembly

A kmer analysis was performed to estimate the genome size, level of heterozygosity and repeat content of the sequenced genome. Using the software Jellyfish v1.1.10 [3], 17-mers were extracted from the WGS PE reads (PE400), and unique kmers were counted and plotted according to kmer depth (Fig. 1). The homozygous or main peak is found at a depth of ~52x. The estimated genome size (found by dividing the total number of kmers by the kmer depth of the main peak) is 1.38 Gb, which is at the low end of the range of empirical estimates. The C-value ranges from 1.45–2.33 pg (1.42 Gb–2.28 Gb), with the median at 1.59 pg (1.56 Gb) (data from [4], see [5–9]), suggesting the existence of variation in the repetitive fraction of the genome for the species. The left peak at 26x kmer depth indicates many polymorphic sites in the genome. In fact, using the Genomic Character Estimator program, gce v 1.0.0 [10], the heterozygous ratio based in kmer individuals is 0.054, and the corrected estimate of genome size is 1.32 Gb. Hereon the gce estimate is referred to as the ‘assemblable’ portion of the genome.

A pilot WGS assembly using only PE data was performed in order to generate enough contiguous sequences to gather library insert size statistics. PE reads were first filtered for contaminating sequences (phiX, *Escherischia coli* and other vector sequences, as well as *O. europaea* plastids) using GEM [11] with –m 0.02 (2 % mismatches). Then, the reads were assembled into scaffolds using AbySS v1.3.6 [12] with parameters: −s 600 − S 600–3000 − n 6 − N 10 − k 127 − l 75 − aligner map − q 10. This resulted in an assembly with a total length of 1.94 Gb, and contig and scaffold N50s of 3.7 kb and 3.8 kb, respectively. Library insert sizes were estimated by mapping against this draft assembly. For the WGS PE libraries sequenced on Illumina HiSeq2000 using 2x151 bp reads, the insert size distribution followed a bimodal distribution with a main peak at 725 bp and a smaller peak at 300 bp. Before continuing with the assembly, read pairs belonging to the smaller peak were filtered out, if connecting reads were found overlapping both mates of the pair.

**Fig. 2:**
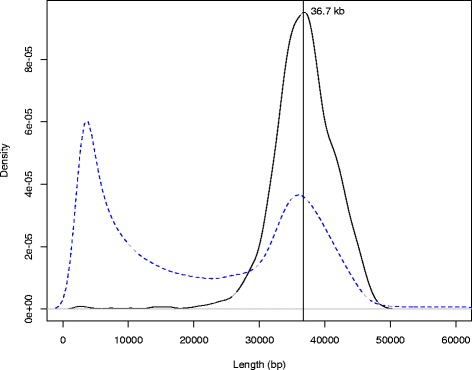
Comparison of fosmid insert and fosmid-pool scaffold size distributions. Fosmid clone insert size estimates (black contiguous line) were obtained by mapping fosmid end sequences to our merged fosmid pool (FP) assembly. The fosmid end sequencing of only 155,000 unique clones resulted in a very high sequencing depth, so we set a lower threshold of 100 x for the number of times a given length was seen and counted each length only once. While this procedure results in underestimating the amplitude of the density peak, both the shape of the distribution and the mean insert size (36.7 kb) should be unaffected, while the standard deviation is likely an overestimate. The distribution of scaffold lengths from the 96 fosmid pool assemblies is given by the blue dashed line (scaffolds smaller than 2.5 kb were discarded to avoid noise)

**Fig. 3:**
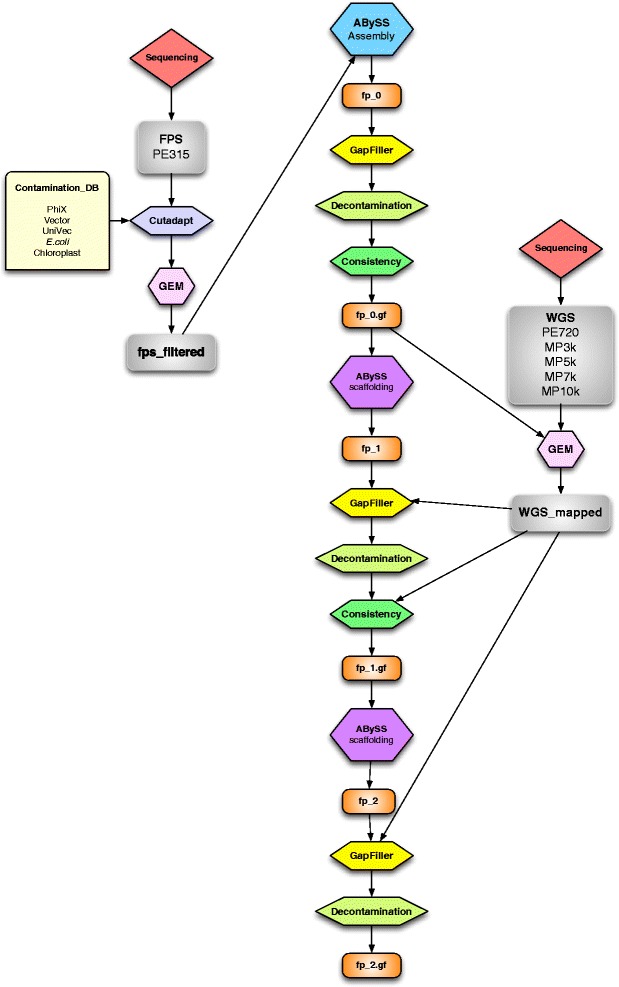
Fosmid pool assembly pipeline. For each fosmid pool, a single paired-end (PE) library sequenced at 2 x 150 bp was first filtered and trimmed of pNGS vector sequences, as well as those of *Escherichia coli* and other common contaminants, including *Olea europaea* chloroplast sequences. Reads were assembled with ABySS, gapfilled with GapFiller, and contaminants removed using a BLAST homology search. A consistency check was performed, breaking the assemblies at any point inconsistent with the proper insert size and orientation of fosmid pool PE reads. The resulting contigs were scaffolded using whole genome shotgun (WGS) data, followed by another round of gapfilling, decontamination and consistency checking, this time including the new WGS data. To repair the consistency broken assembly, a final round of scaffolding, gapfilling and decontamination was performed

The inflated length (47 % of the assemblable part of the genome) and the poor contiguity obtained for the draft assembly are symptomatic of the expected difficulty in distinguishing divergent alleles of the same locus from true repeats. To address this challenge, the 96 sequenced fosmid pools (3.9x physical coverage of the genome, each pool covering ~4 % of the genome) were assembled using the assembly pipeline shown in Fig. 2 to obtain 96 largely haploid assemblies (simulations of 1,600-clone pools with a genome size of 1.38 Gb show a mean of 2.5 % of sequenced bases to derive from separate overlapping clones, half of which would come from different alleles). Optimal kmer size was 97 for most of the pools. For each pool a base assembly was produced using ABySSv1.3.7 and parameters: −s 300 − S 300–5000 − n 9 − N 15 − k 97 − l 75 − aligner map − q 10. Afterwards, the base assemblies went through several rounds of gapfilling [13], decontamination, consistency checks, and rescaffolding with ABySSv1.3.7. The decontamination step consists of detecting contaminant sequences (phiX, vectors, UniVec, *E. coli*, plastids) in the intermediate assemblies using *blastn* and masking any matches with Ns, thus producing gaps in the assembly. As a result of the FP pipeline, 96 individual assemblies were obtained with an average scaffold N50 of 33,786 ± 3,105 bp. The distribution of scaffold sizes follows a bimodal distribution (Fig. 3), suggesting that a large fraction of fosmid clones are fully assembled. Mapping of fosmid ends to the merged assembly (‘FP assembly’, see below) gives an estimate of the clone insert size distribution (mean of 36.7 kb ± SD 4.97 kb) that corresponds well with the right peak of the scaffold sizes.

**Fig. 4:**
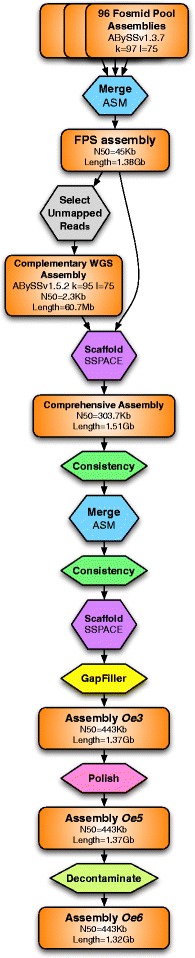
Overview of the complete assembly pipeline. The basic flow chart starting with the 96 fosmid pool assemblies is shown. Assemblies are shown in orange rounded rectangles. All computational steps are shown as octagons

The 96 fosmid pool assemblies were then merged based on overlaps using in-house OLC-like assembly-merging software called ASM (L. Frias and P. Ribeca, manuscript in preparation; scripts are publicly available at [14]. Two rounds of merging were performed, with intermediate scaffolding and gapfilling steps. In the first round, a minimum overlap of 2,400 bp and high sequence similarity (maximum edit distance of 1.5 %) was used, while in the second round, longer overlaps (4,000 bp) and higher sequence divergence (maximum edit distance of 10 %) were used in order to merge allelic regions. Each round of merging collapses repeats unless higher order information supports a unique path for resolving a repetitive region; this includes both the sequence of the input data (contigs) and scaffolding information (i.e., the order of contigs in scaffolds in the original fosmid pool assemblies). Merging produced an intermediate assembly (named ‘FP assembly’ in Fig. 4) with a scaffold N50 of ~45 kb and a total length of 1.38 Gb. Although this assembly was 4.54 % larger than the assemblable genome size (1.32 Gb), gene completeness according to CEGMA was only 95.97 % complete and 97.58 % partial, suggesting that 2.42–4.03 % of the gene space may have been missed.

To increase the overall completeness of the assembly, all WGS reads that did not map to the FP assembly were selected and used to obtain a complementary assembly using ABySSv1.5.2 with parameters: −s 300 − S 300–5000 − n 10 − N 10 − k 95 − l 75 − aligner map − q 10. This assembly accounts for 60.7 Mbp of sequence, and has an N50 of 1,506 bp for contigs and 2,351 bp for scaffolds. This assembly was then broken into contigs, 50 bp was eroded from the ends of each contig, then contigs smaller than 200 bp were filtered out. Both assemblies were subsequently gathered by joining the WGS contigs with the merged fosmid pool assembly, and scaffolding them with SSPACE 2.0 [15]. To account for read pairs coming from two different alleles in the same genomic region, reads were mapped to the SSPACE input assembly with gem-mapper (settings: m = 0.05 and e = 0.1) and filters were applied to detect unique mappings with no subdominant match. The resulting comprehensive assembly had a scaffold N50 of 303.7 kb and a total length of 1.51 Gb, ~190 Mb above the expected genome length (1.32 Gb). The excess of assembled sequence is likely to be caused by the presence of artificial duplications during the assembly process (i.e., uncollapsed haplotypes that have been resolved in two different contigs). Several strategies were used to refine the assembly and obtain a haploid reference. First, consistency check was applied to remove local misassemblies by mapping short and intermediate libraries (PE720, MP3k and MP5k) to the input assembly: a positive score is assigned to the assembly regions supported by read pairs separated by distances falling within the limits (mean ± 3σ) of the empirical distribution, while a negative score is assigned to regions where read pairs map i) outside of these bounds, ii) in inconsistent orientation, or iii) to different scaffolds. Regions where the sum of these two vectors is negative are removed from the assembly. After applying this consistency check, the resulting assembly had 46,893 consistent contig blocks (compared to 25,042 contigs before the consistency check), giving a total of 1.46 Gb with an N50 of 101 kb. Second, this assembly was collapsed using a minimum overlap of 4 kb and the gem-mapper parameters − e 0.03 and − m 0.02, so only close matches were merged (similar uncollapsed haplotypes, identical assembly artifacts, and near identical repeats). Additionally, in order to avoid spurious joins, tip merging was applied to the alignment graph down to overlaps of 250 bp. Finally, no repeat resolution was applied, but coherent links from input scaffolds were reinserted. Consequently, the assembly length shrunk to ~1.30 Gb, almost matching the assemblable fraction of the genome (1.32 Gb). An additional consistency check was run on the collapsed assembly using the short and intermediate libraries (PE720, MP3k and MP5k), which resulted in breaking the assembly from 64,814 into 72,593 scaffolds, giving a total length of 1.30 Gb with a scaffold N50 of 50 kb. This assembly length is what was expected based on the gce estimate. As a final assembly step, PE reads with high divergence (gem-mapper parameters m = 0.05 and e = 0.08) were mapped to the assembly and rescaffolded with SSPACE 2.0 using parameters k = 3 and a = 0.6. Then, scaffolds shorter than 500 bp were discarded, and the GapFiller program [13] was used to close about 40 % of the assembly gaps. This assembly was labeled ‘Oe3’.

The Oe3 assembly was polished using a mapping-based strategy designed to correct single nucleotide substitution and short insertion–deletion errors. First, one library of paired-end reads (PE725) was aligned using BWA mem (v0.7.7) [16] and variant calling was performed. Selecting only homozygous alternative variants, an alternative FASTA sequence was obtained using GATK (v3.5) FastaAlternateReferenceMaker [17]. After discarding scaffolds shorter than 500 bp, the resulting assembly (Oe5) had a scaffold N50 of 444 kb and a contig N50 of 51 kb. After detecting putative contamination in some scaffolds of the Oe5 assembly, a final decontamination step was performed against yeast, bacteria, arthropod and mitochondrial sequences, combining homology search results obtained by BLAST and, in the case of mitochondrial sequences, regions of high depth (~6000x). In total, 509 scaffolds were deleted from Oe5 and some parts of another 27 scaffolds were removed. The assembly resulting from this step, Oe6, has a scaffold N50 of 443 kb and a contig N50 of 52 kb (Table 2). Oe6 contains 48,419 gaps comprising 53,969,601 sites. The gene completeness of this assembly was estimated using CEGMA [18] and BUSCO (Benchmarking Universal Single-Copy Orthologs) [19]. CEGMA analysis resulted in a gene completeness of 98.79 %, while BUSCO, using a plant-specific database of 956 genes, determined a completeness of 95.6 % of plant genes. A summary of the complete assembly strategy is shown in Fig. 4.

### Partial assembly of an olive tree associated fungus: *Aureobasisium pullulans*

One of the putative sources of non-plant sequence present in the olive samples was considered of interest; it was represented among the fosmid pools and seemed to belong to the fungal genus *Aureobasidium*, which has been previously associated with olive trees [20]. To assemble a partial sequence of this genome, four fully sequenced *Aureobasidium* genomes were downloaded from JGI [21]. Then, BWA v0.7.3a [16] was used to map all the reads from the fosmid libraries to the four genomes. Once mapped, the reads were filtered allowing only soft clipping for a maximum of one-third of the read, and deleting read pairs when only one of the pairs passed the filters. This resulted in a collection of 18,549,090 reads, which were assembled with SPAdes v.3.1.1 [22]. Scaffolding was done using the assembled fosmids using SSPACE-LongRead [23], and gaps were filled with gapcloser [24]. These two steps were repeated twice. The final alignment was then compared to the *Aureobasidium* genomes using BLAST. Contigs longer than 200 nt, for which less than 20 % of their sequence mapped against any of the *Aureobasidium* genomes, were separated and compared against the NCBI non-redundant nucleotide database [25]. Only those contigs with first hits to fungal species were kept. The final assembly comprised 18 Mb, roughly two-thirds of the typical size of *Aureobasidium* genomes (25–29 Mb). To identify the species and strain, the most common fungal markers used for fungal barcoding were identified (ITS, SSU, LSU, RPB1, RPB2 and EF1). Most of the markers were missing in the assembly or were too short; based on a 769 nt fragment of the RPB1 gene, the most similar sequence was that of *Aureobasidium pullulans* isolate AFTOL-ID 912 (DQ471148.1); a strain that was isolated from the grape plant *Vitis vinifera*. The identity of this fragment was 99.95 % indicating that this was likely a different strain of the same species. Augustus [26] was used to perform gene annotation. The training parameters were obtained using scaffold 1 of the published *A. pullulans* genome, and then used to predict proteins in our strain of *A. pullulans*. This resulted in 6,411 proteins.

### Olive tree genome annotation

**Fig. 5:**
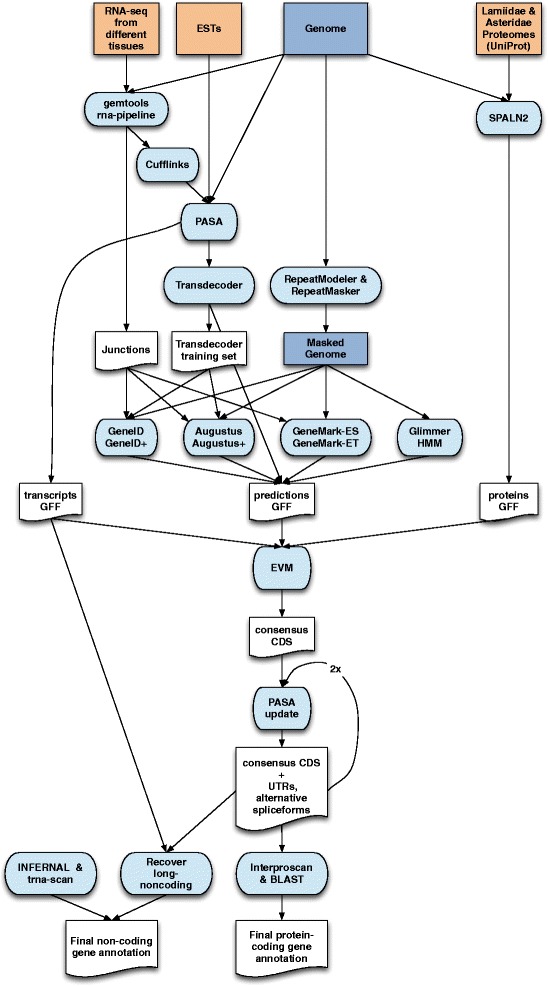
Overview of the annotation pipeline. Input data for annotation are shown at the top of the flow chart. Computational steps are shown in light blue and intermediate data are shown in white

To annotate the olive tree genome, consensus gene models were obtained by combining transcript alignments, protein alignments, and gene predictions. A flowchart outlining these steps is shown in Fig. 5. Transcripts for assembly with Program to Assemble Spliced Alignments (PASA; r2014-04-17) [27] were obtained as follows: first, RNA-Seq reads generated from different tissues by our group (see above), plus publicly available datasets in the Sequence Read Archive (SRA) (Table 3), were aligned to the final assembly Oe6 with GEM v1.6.1 [11]. Transcript models were subsequently generated using the standard Cufflinks v2.1.1 pipeline [28] – starting with the BAM files, resulting in 2,056,606 transcripts, which were then added to the PASA database. In addition, 12,959 olive expressed sequence tags (ESTs) and mRNAs present in Genbank (October 27, 2014) [29–31] were also added to PASA using GMAP v2013-10-28 [32] as the alignment engine. All of the above transcript alignments were then assembled by PASA, resulting in 942,302 PASA assembled transcripts, which were scanned with PASA’s Transdecoder program [27] to detect likely protein coding regions. This tool predicted a total of 169,562 candidate genes. From these, a training set for *ab initio* gene predictors was created from PASA models coding for complete proteins, longer than 500 amino acids and with a BLAST hit to either the *Lamiidae* or *Asteridae* proteomes. A training set of 589 non-redundant genes was obtained. In addition, the complete *Lamiidae* and *Asteridae* proteomes present in Uniprot (February 10, 2015) were aligned to the olive genome using SPALNv2.1.2 [33], resulting in 625,980 coding sequence (CDS) alignments.

**Table 3:** RNA-Seq samples used for annotation

Accession	Tissue	Varietal
ERS1146989	Immature olives	Farga
ERS1146988	Roots	Farga
ERS1135096	Old leaves	Farga
ERS1135095	Young leaves	Farga
ERS1135094	Flowers	Farga
ERS1135093	Flower buds	Farga
ERS1135092	Green olives	Farga
SRP000653	Fruits	Coratina
SRP005630	Buds	Picual, Arbequina
SRP044780	Leaves, Roots	Picual
SRP016074	Fruits, leaves, stems and seeds	Picula x Arbequina
SRP017846	Fruits	Istrska belica
SRP024265	Leaves, Roots	Kalamon

For *ab initio* gene prediction, transposable element repeats in the Oe6 assembly were first masked with RepeatMasker v4-0-5 [34] using a custom repeat library constructed by running RepeatModeler v1-0-7 and adding some olive-specific repeats [35]. A search was also carried out for masked proteins encoded by transposable elements (TEs) provided in the RepeatMasker Library of TE proteins. Low complexity repeats were left unmasked for this purpose. In total, 63 % of the assembly was masked.

On this masked assembly four different *ab initio* gene predictors were run, since combiners like EvidenceModeler work better when finding consensus among the output of a diverse set of gene prediction algorithms, and orthogonal evidence such as transcript and protein mapping. *O. europaea* protein-coding gene predictions were obtained with GeneID v1.4.4 [36] trained specifically for *O. europaea* with GeneidTrainer using the training set of 589 genes; with Augustus v3.0.2 [26] trained with the *etraining* script that comes with Augustus using the same training set; and with GlimmerHMM v3.0.1 [37] trained with the *trainGlimmerHMM* script that comes with the program using the same training set. Finally, GeneMark-ES v2.3 [38] gene predictions were obtained by running it in its self-trained mode. The number of predicted gene models ranged from 48,237 with GeneMark-ES to 97,542 with GlimmerHMM. Geneid, Augustus and Genemark-ET v4.21 were also used to generate predictions incorporating intron evidence, which was extracted from the RNA-Seq data, by obtaining the junctions after mapping it with GEM (see below). Junctions overlapping with *ab initio* GeneID predictions, Augustus predictions, or with protein mappings were taken as intron evidence. Running GeneID with hints resulted in a total set of 74,231 gene models; Augustus with hints resulted in 70,906; and Genemark-ET with 64,329 gene models.

Evidence Modeler r2012-06-25 (EVM) [39] was used to obtain consensus CDS models using the three main sources of evidence described above: gene predictions, aligned transcripts and aligned proteins. EVM was run with three different sets of evidence weights, and the resulting consensus models with the best specificity and sensitivity as determined by intersection (BEDTools v2.16.2 intersect [40]) with the transcript mappings, were chosen for the final annotation (Table 4 shows the best-performing weights). Consensus CDS models were then updated with untranslated regions (UTRs) and alternative exons through two rounds of PASA annotation updates. A final quality control was performed to fix reading frames and intron phases, and remove some transcripts predicted to be subject to nonsense-mediated decay. The resulting transcripts were clustered into genes using shared splice sites or substantial sequence overlap as criteria for designation as the same gene. This resulted in a preliminary set of 56,349 protein-coding genes, whose 89,982 transcripts encode 79,910 unique protein products (~1.59 transcripts per gene). Systematic identifiers with the prefix ‘OE6A’ were assigned to the genes, transcripts and derived protein products. Functional annotation was performed with InterProScan-5.17-56.0 [41], 30,900 protein-coding genes were annotated with gene ontology (GO) terms, and 41,257 were assigned a function.

**Table 4:** Weights given to each source of evidence when running Evidence Modeler r2012-06-25

Type of evidence	Program	Weight
ABINITIO_PREDICTION	GeneMark	1
ABINITIO_PREDICTION	Augustus	1
ABINITIO_PREDICTION	geneid_v1.4	1
ABINITIO_PREDICTION	GlimmerHMM	1
ABINITIO_PREDICTION	geneid_introns	2
ABINITIO_PREDICTION	Augustus_introns	2
ABINITIO_PREDICTION	GeneMark-ET	2
OTHER_PREDICTION	transdecoder	2
TRANSCRIPT	PASA	10
PROTEIN	SPALN	10

**Fig. 6:**
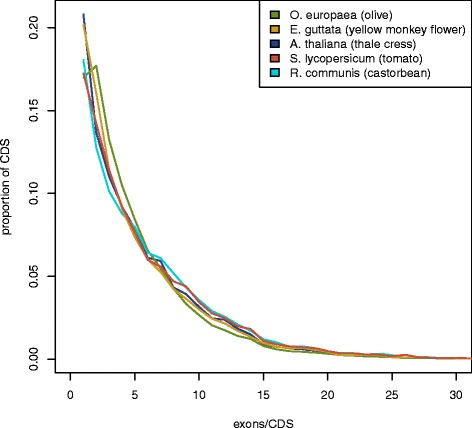
Distribution of exons per coding sequence in the analyzed species. The number of exons per CDS feature (UTRs were ignored) was counted and the distribution plotted for the olive and each of the other four species for which we compared annotations. Similar distributions were observed for all species

The predicted *O. europaea* protein-coding set was then compared with those in four other selected plant genomes (*Arabidopsis thaliana*, *Erythranthe guttata*, *Solanum lycopersicum*, and *Ricinus communis*) downloaded from the NCBI database. A BLASTP search of those proteomes was also performed against the olive proteome, and vice versa, using the BLASTALL 2.2.25+ software suite [42] with an e-value less than 0.01 and with at least 50 % of identity (Table 5). General statistics for transcript, coding sequence and exon lengths in *O. europaea* are similar to those in the other species, but the number of genes is significantly larger. The number of exons per transcript is slightly lower than in the four compared species. It is possible that more false-positive single-exon genes have been annotated; however, the number of single-exon CDS is not higher, although there is a slight shift in the distribution toward fewer coding exons per transcript (Fig. 6).

**Table 5:** Comparison of *O. europaea* with other plant species

Species	Number of proteins	Average transcript length (bp)	Average coding sequence length (bp)	Average exons per transcript	Average exon length (bp)	Proteins with homologs in *O. europaea*	*O. europaea* proteins with homologs in the other species
*Olea europaea*	56,349	3,953	1,050	4.54	315	56,349 (100 %)	56,349 (100 %)
*Arabidopsis thaliana*	35,378	2,341	1,234	5.89	261	23,106 (65.3 %)	32,796 (58.2 %)
*Erythranthe guttata*	31,861	3,378	1,351	5.77	300	24,373 (76.5 %)	42,458 (75.3 %)
*Solanum lycopersicum*	36,148	5,626	1,389	6.48	288	27,778 (76.8 %)	38,448 (68.2 %)
*Ricinus communis*	27,998	4,323	1,390	6.53	287	21,990 (78.5 %)	37,264 (66.1 %)

Average of the transcript length, coding sequence, exons per transcript and exon length of *O. europaea*, *Arabidopsis thaliana*, *Erythranthe guttata*, *Solanum lycopersicum* and *Ricinus communis* proteomes, the number of proteins with at least one homolog in *O. europaea* and the number of proteins of *O. europaea* with at least one homolog in the other species. The longest protein isoform per gene was used for homology search

The increased number of coding genes in *O. europaea* suggests the existence of a large-scale genome duplication with respect to the other species. Although this possibility deserves more detailed analysis, preliminary analyses of gene comparisons identified 34,195 *O. europaea* genes with *O. europaea* paralogs that are more similar to each other than to the corresponding best hit in *E. guttata* (80.5 % of the total proteins with hits in *E. guttata*), the closest species in this analyses. Also, from the 14,437 paralogous pairs found in *O. europaea* that represent each other’s reciprocal best hit, 10,711 pairs had the same best hit in *E. guttata* (which represents 74.2 % of the pairs). These results suggest that a high proportion of the *O. europaea* gene repertoire has been duplicated since the separation of these two lamiales species. To discard the possibility that these duplicates resulted from uncollapsed heterozygous alleles, heterozygous single nucleotide variants (SNVs) identified by variant calling using samtools mpileup in pairs of putatively recent duplicates were counted and compared with those in singletons (genes without recent paralogs). The mean is significantly higher in genes within recent duplicate pairs (Welch’s Two Sample *t*-test *p*-value < 2.2e-16). Finally, the 70 % quantile of two-copy SNV counts is 42 and 8 for the one-copy genes. In the case where uncollapsed (duplicated) alleles are frequent, one would expect to obtain the opposite pattern, as reads coming from the same locus would independently map to one of the two uncollapsed haplotypes in the assembly, thus dramatically reducing the number of heterozygous SNVs called. Although further and more detailed analyses are required, these results suggest extensive gene duplication in the lineage leading to the olive tree. The possibility of a whole genome duplication is consistent with the increased chromosomal number in *O. europaea* (2n = 46), as compared to closely related lamiales such as *Erythranthe guttata* (2n = 28) [43] and *Sesamum indicum* (2n = 26) [44].

Non-coding RNAs (ncRNAs) were annotated by running the following steps. First, the program cmsearch (v1.1) that comes with Infernal [45] was run with the Rfam database of RNA families (v12.0) [46]. Also, tRNAscan-SE (v1.23) [47] was run in order to detect the transfer RNA genes present in the genome assembly. To detect long non-coding RNAs (lncRNAs), PASA assemblies that had not been included in the annotation of protein-coding genes (i.e., expressed genes that were not translated to protein) were first selected. Those longer than 200 bp and with a length not covered by a small ncRNA at least 80 % were incorporated into the ncRNA annotation as lncRNAs. The resulting transcripts were clustered into genes using shared splice sites or significant sequence overlap as criteria for designation as the same gene. Systematic identifiers with the prefix ‘OE6ncA’ were assigned to the genes and their derived transcripts. In total, 25,199 non-coding genes have been annotated, among which 20,082 are lncRNAs.

In summary, we report the first genome sequencing, assembly, and annotation of the Mediterranean olive tree. This genome assembly will provide a valuable resource for studying developmental and physiological processes, investigating the past history of domestication, and improving the molecular breeding of this economically important tree.

## Abbreviations

CDS, coding sequence(s); ENA, European Nucleotide Archive; EST, expressed sequence tag; EVM, Evidence Modeler r2012-06-25; FP, fosmid pools; Gb, gigabase; GO, Gene Ontology; lncRNA, long non-coding RNA; MP, mate-pairs; ncRNA, non-coding RNA; PASA, Program to Assemble Spliced Alignment; PE, paired-end; pg, picograms; SNV, single nucleotide variant; SRA, Sequence Read Archive; TE, transposable element; UTR, untranslated region; WGS, Whole Genome Shotgun
